# SIK2 Drives Pulmonary Fibrosis by Enhancing Fibroblast Glycolysis and Activation

**DOI:** 10.3390/biomedicines13081919

**Published:** 2025-08-06

**Authors:** Jianhan He, Ruihan Dong, Huihui Yue, Fengqin Zhang, Xinran Dou, Xuan Li, Hui Li, Huilan Zhang

**Affiliations:** 1 National Health Commission Key Laboratory of Respiratory Diseases, Department of Respiratory and Critical Care Medicine, Tongji Hospital, Tongji Medical College, Huazhong University of Science and Technology, Wuhan 430030, China; hejianhan1234@163.com (J.H.); dongruihan1998@126.com (R.D.); m15002760104@163.com (H.Y.); fengqinz96@163.com (F.Z.); ddddxr1018@163.com (X.D.); 13387634530@163.com (X.L.); lihui010904@163.com (H.L.); 2 Department of Respiratory and Critical Care Medicine, Ningbo No. 2 Hospital, Ningbo 315100, China; 3 State Key Laboratory for Diagnosis and Treatment of Severe Zoonotic Infectious Disease, Huazhong University of Science and Technology, Wuhan 430030, China

**Keywords:** pulmonary fibrosis, fibroblasts, metabolic reprogramming, SIK2

## Abstract

**Background:** Pulmonary fibrosis (PF), the end-stage manifestation of interstitial lung disease, is defined by excessive extracellular matrix deposition and alveolar destruction. Activated fibroblasts, the primary matrix producers, rely heavily on dysregulated glucose metabolism for their activation. While Salt Inducible Kinase 2 (SIK2) regulates glycolytic pathways in oncogenesis, its specific contributions to fibroblast activation and therapeutic potential in PF pathogenesis remain undefined. This study elucidates the functional role of SIK2 in PF and assesses its viability as a therapeutic target. **Methods:** SIK2 expression/localization in fibrosis was assessed by Western blot and immunofluorescence. Fibroblast-specific Sik2 KO mice evaluated effects on bleomycin-induced fibrosis. SIK2’s role in fibroblast activation and glucose metabolism impact (enzyme expression, metabolism assays, metabolites) were tested. SIK2 inhibitors were screened and evaluated therapeutically in fibrosis models. **Results:** It demonstrated significant SIK2 upregulation, specifically within activated fibroblasts of fibrotic lungs from both PF patients and murine models. Functional assays demonstrated that SIK2 is crucial for fibroblast activation, proliferation, and migration. Mechanistically, SIK2 enhances fibroblast glucose metabolism by increasing the expression of glycolysis-related enzymes. Additionally, this study demonstrated that the SIK2 inhibitor YKL06-061 effectively inhibited PF in both bleomycin and FITC-induced PF mouse models with the preliminary safety profile. Furthermore, we identified a novel therapeutic application for the clinically approved drug fostamatinib, demonstrating it inhibits fibroblast activation via SIK2 targeting and alleviates PF in mice. **Conclusions:** Our findings highlight SIK2 as a promising therapeutic target and provide compelling preclinical evidence for two distinct anti-fibrotic strategies with significant potential for future PF treatment.

## 1. Introduction

Interstitial lung disease (ILD) encompasses conditions affecting the lung’s interstitial tissue, causing inflammation, edema, or fibrosis, which results in impaired gas exchange [1,2]. When ILD primarily involves fibrotic changes, it is termed fibrosing interstitial lung disease (F-ILD) or pulmonary fibrosis (PF), regarded as the final stage of ILD [3]. PF diseases include idiopathic pulmonary fibrosis (IPF), interstitial lung disease associated with connective tissue diseases (CTD-ILD), hypersensitivity pneumonitis, silicosis, asbestosis, etc. PF involves alveolar damage and excessive extracellular matrix (ECM) buildup, reducing lung flexibility and impairing gas exchange, causing irreversible changes in lung tissue [4]. Symptoms include dry cough and dyspnea, with severe cases potentially leading to respiratory failure or death. The clinical drug treatment options available for PF are very limited, and only two FDA-approved drugs are available for PF treatment currently [5]. Understanding the mechanisms of PF is essential for developing effective anti-fibrotic drugs [6].

Fibroblasts, as the main ECM source, are key to PF progression, making their activation a crucial target for PF treatment [7]. During the progression of PF, pro-fibrotic cytokines (e.g., TGF-β and PDGF-α/β) in the microenvironment of lung tissue transform fibroblasts into myofibroblasts that increase ECM secretion, proliferation, and migration, resulting in excessive ECM deposition and fibrotic foci [8]. Research has indicated that fibroblast activation is modulated by a range of mechanisms, including mechanical force, endoplasmic reticulum stress, and pro-fibrotic pathways [9]. Recently, the pivotal role of metabolic reprogramming has garnered significant attention. Alterations in glucose, lipid, and amino acid metabolism, alongside mitochondrial dysfunction, have been observed during the progression of PF, with particularly pronounced changes noted in glucose metabolism [10,11]. Research has demonstrated that patients with IPF exhibit increased glycolytic activity within fibrotic regions, and glycolytic capacity has been recognized as a predictive marker for reduced lung function and elevated mortality rates [12,13]. Subsequent mechanistic studies have elucidated the pivotal regulatory role of glucose metabolism reprogramming in fibroblast activation, underscoring its potential as a therapeutic target for modulating fibroblast activity and alleviating pulmonary fibrosis [14,15]. Nonetheless, there are currently no anti-fibrotic therapies available that specifically target fibroblast glucose metabolism.

The AMP-activated Protein Kinase (AMPK) family is a member of serine/threonine protein kinases and is highly conserved during evolution. Its activator is AMP, a product of ATP hydrolysis, making it highly sensitive to energy metabolism [16,17,18]. Salt Inducible Kinase 2 (SIK2), a constituent of the AMP-activated protein kinase family, is integral to the regulation of cellular metabolism. Its role in modulating glucose metabolism has been substantiated in various oncological studies [19,20]. Research indicates that the knockdown of SIK2 in colorectal and ovarian cancer cells results in a 50% reduction in cellular glucose metabolism, underscoring SIK2’s significant regulatory influence on this metabolic pathway [21,22]. Regarding the role of SIK2 in the development of PF, existing studies have shown that a SIK2 inhibitor (ARN-3236) can inhibit bleomycin (BLM)-induced PF in mice [23]. However, there is no definite evidence to show whether SIK2 mediates the occurrence of PF by promoting fibroblast activation, and the molecular mechanism of its action remains unclear.

In this study, we generated fibroblast-specific *Sik2*-knockout mice to investigate the role of Sik2 in fibroblasts under conditions of PF. Our results demonstrated that SIK2 is integral to the regulation of glucose metabolism in fibroblasts, which in turn influences the activation process of these cells. Additionally, we examined the potential of SIK2 as a therapeutic target for the treatment of PF.

## 2. Material and Methods

### 2.1. Reagents and Antibodies

Bleomycin, YKL06-061, SB431542, and fostamatinib were procured from MedChemExpress (Monmouth Junction, NJ, USA, catalog numbers: HY-A0281, HY-120056, HY-10431, and HY-13038A). Recombinant TGF-β1 was sourced from PeproTech (Cranbury, NJ, USA, catalog number: 100-21). Antibodies targeting fibronectin, SIK2, HK2, and LDHA were obtained from Abclonal Biotechnology Co., Ltd. (Wuhan, China, catalog numbers: A12932, A8321, A22319, and A21893). The Phospho-SIK2 (Thr175 and Thr163) polyclonal antibody was acquired from Invitrogen (Carlsbad, CA, USA, catalog number: PA5-64607). Antibodies against β-actin, collagen 1, PGAM1, PFKFB3, and PKM2 were sourced from Proteintech Group, Inc. (Wuhan, China, catalog numbers: 66009-1-Ig, 68288-1-Ig, 16126-1-AP, 13763-1-AP, and 15822-1-AP), while antibodies for α-SMA were obtained from Cell Signaling Technology (Boston, MA, USA, catalog number: #19245).

### 2.2. Experimental Animals and the Pulmonary Fibrosis Mouse Model

Wild-type C57BL/6 mice (male, aged 6–8 weeks), *Col1a2-CreERT2* mice, and *Sik2-loxp* mice were acquired from Beijing Vital River Laboratory Animal Technology Co., Ltd (Beijing, China). All experimental animals were housed under specific pathogen-free conditions at the Caidian Research Building Animal Center, Tongji Hospital. Mice were anesthetized using isoflurane and subsequently administered BLM intratracheally at a dose of 1.8 mg/kg to establish a PF mouse model, as previously described [24]. On the 21st day post bleomycin treatment, the mice were euthanized, and lung tissues were harvested for analysis. In experiments involving the use of small-molecule inhibitors for the treatment of PF, YKL-06-062 (2 mg/kg, intraperitoneal injection administration) and fostamatinib (20 mg/kg, intragastric administration) were administered to the mice on the 12th, 14th, 16th, 18th, and 20th days following BLM injection. The control group received the same administration protocol with an equivalent volume of solvent. Pirfenidone (200 mg/kg, intragastric administration), serving as a positive control, was administered daily from the 12th to the 20th day. In the FITC-induced pulmonary fibrosis mouse model, mice were anesthetized using isoflurane and subsequently received an intratracheal administration of FITC at a dosage of 20 mg/kg. All other experimental conditions were consistent with those employed in the BLM-induced pulmonary fibrosis mouse model. YKL-06-062 and fostamatinib were solubilized in PBS with the addition of 5% DMSO, while pirfenidone and BLM were dissolved solely in PBS. *Col1a2-CreERT2* mice need to be intraperitoneally injected with tamoxifen (75 mg/kg) for five consecutive days starting 12 days before BLM treatment to induce gene knockout (Figure S1).

### 2.3. Human Samples

Fibrotic lung samples were obtained from patients with PF who underwent lung transplantation, including five cases of IPF and two cases of CTD-ILD. In contrast, normal control lung samples were procured from para-carcinoma tissues excised during lung cancer resections (n = 5, at a minimum distance of 5 cm from the malignant tissue). Informed consent was obtained from all research participants. All diseases are diagnosed in accordance with the latest diagnostic criteria. All procedures involving human subjects received approval from the Medical Ethics Committee (TJ-IRB20231297). Table S1 provides a comprehensive overview of the baseline characteristics of participants who contributed lung tissue samples.

### 2.4. Human Precision-Cut Lung Slices

The preparation of human precision-cut lung slices (HPCLS) and subsequent treatment were conducted following established protocols [24]. Briefly, the lung tissue was washed with PBS, infused with 2% agarose in PBS, and solidified in cold PBS. It was then cut into 400 µm slices using a vibrating blade microtome. These slices were washed with PBS and cultured in DMEM with 0.5% fetal bovine serum and antibiotics. After 48 h, a fibrosis-inducing cocktail comprising 20 ng/mL TGF-β1 (catalog number 100-21, Peprotech, Wuhan, China), 20 ng/mL PDGF-AB (catalog number 100-00AB, Peprotech), 10 ng/mL TNF-α (catalog number P06804, R&D Systems, Minneapolis, MN, USA), and 5 μM 1-Oleoyl lysophosphatidic acid (catalog number 62215, Cayman Chemical, Ann Arbor, MI, USA) was applied. Subsequently, the tissues were harvested after the 48 h stimulation period for further analysis.

### 2.5. Primary Lung Fibroblast Culture and Treatment

According to the protocol described in previous studies, primary lung fibroblasts were extracted from healthy human lung tissues or lung tissues of mice [25]. The fibroblasts were cultured in DMEM supplemented with 10% fetal bovine serum. Activation of the fibroblasts was induced by treating the cells with recombinant TGF-β1 at a concentration of 10 ng/mL.

### 2.6. Histological and Immunofluorescence Analysis

The lung tissues of mice were initially perfused with neutral tissue fixative (Servicebio, Wuhan, China) and subsequently immersed in paraformaldehyde for an additional 24 h to ensure thorough fixation. Following fixation, the lung tissues were subjected to dehydration, paraffin embedding, and sectioning into slices with a thickness of 5 µm. These sections were then stained using H&E, Sirius Red, and Masson’s trichrome stains. For the immunofluorescence analysis, paraffin sections were deparaffinized and subjected to antigen retrieval. Immunostaining was subsequently conducted using antibodies specific to fibronectin, collagen 1, SPC, CD68, or α-SMA. Following this, anti-rabbit/mouse antibodies conjugated with Alexa Fluor 488/594 (Servicebio, Wuhan, China) were applied. The sections were then counterstained using a DAPI staining kit (Servicebio, Wuhan, China). Finally, fluorescence images were acquired utilizing a fluorescence microscope (Olympus Corporation, Tokyo, Japan).

### 2.7. Western Blot Analysis

The methodology previously described was utilized for the analysis conducted via Western blot [24]. In summary, protein samples were prepared by homogenizing cultured cells and lung tissues. These samples were then subjected to Western blot analysis utilizing primary antibodies and horseradish peroxidase-conjugated secondary antibodies alongside an enhanced chemiluminescence kit (Biosharp, Beijing, China). Image acquisition was conducted using the ChemiDoc XRS Chemiluminescence Gel Imaging System (Bio-Rad, Berkeley, CA, USA).

### 2.8. Quantitative Real-Time Polymerase Chain Reaction

Total RNA was extracted from the samples utilizing the RNAiso plus reagent (Takara, Kyoto, Japan). The RNA concentration was quantified using a NanoDrop One spectrophotometer (Thermo Fisher Scientific, Waltham, MA, USA). Complementary DNA synthesis was performed with a reverse-transcription kit (Takara, Kyoto, Japan). Subsequent quantitative real-time polymerase chain reaction analysis was conducted using a CFX96 Real-Time PCR Detection System (Bio-Rad, Berkeley, CA, USA). Primers for RT-qPCR are listed in Table S2.

### 2.9. 2-NBDG Glucose Uptake Detection

We adhered to the 2-NBDG Glucose Uptake Assay Kit’s protocol (APExBIO, Houston, TX, USA) to assess glucose uptake by quantifying the fluorescence intensity of the 2-NBDG probe, which retains its fluorescent properties following cellular uptake and phosphorylation. The experiment was initiated once cell confluence in a 96-well black-bottom transparent plate surpasses 80%. Each cell group was subjected to stimulation with specific cytokines and pharmacological agents for a duration of 24 h, subsequently replacing the medium with a glucose-free variant and incubating for 1.5 h. We introduced the 2-NBDG fluorescent probe into the glucose-free medium, replaced the previous medium, and dispensed 100 μL of this new medium into each well, incubating for 20 min. Then, the medium was removed, the cells washed twice with the kit’s Assay Buffer, and 100 μL of Assay Buffer was added to each well, maintaining the samples in darkness for detection. We employed a microplate reader to measure fluorescence intensity at an excitation/emission wavelength of 485/530 nm. The glucose uptake was determined by subtracting the background fluorescence value of the blank well from the fluorescence intensity of the experimental wells.

### 2.10. Statistical Analysis

A quantitative analysis of diverse image files was performed utilizing the ImageJ software (version 1.54g). The results are presented as the mean ± standard deviation. Statistical analyses were executed using the Student’s *t*-test for comparisons between two groups and one-way ANOVA for comparisons involving more than two groups. All analyses and graphical representations were conducted using GraphPad Prism version 8.0 (San Diego, CA, USA). A *p*-value of less than 0.05 was considered to indicate statistical significance.

## 3. Results

### 3.1. SIK2 Protein Level Was Elevated in the Pulmonary Fibrosis Lungs

**Figure 1:**
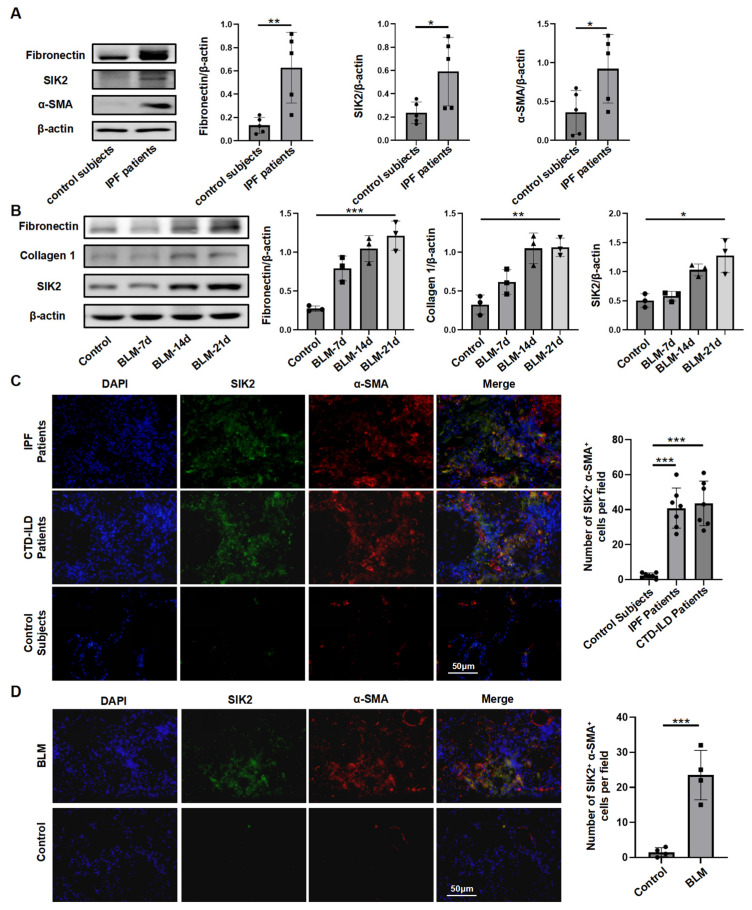
SIK2 protein level was elevated in the pulmonary fibrotic lungs. (**A**) The protein levels of fibronectin, α-SMA, and SIK2 in the lung tissue of IPF patients and control subjects. Left panel: Typical WB images. Right panel: Bar graphs summarizing the levels of each target across different groups. Each group includes five replicates. (**B**) The protein levels of fibronectin, collagen 1, and SIK2 in the lung tissue of PF mice at different time points after BLM treatment. Left panel: Typical WB images. Right panel: Bar graphs summarizing the levels of each target across different groups. Each group includes three replicates. (**C**,**D**) Immunofluorescence images of α-SMA and SIK2 in lung tissue slices of IPF patients, CTD-ILD patients, and BLM-induced PF mice. Left panel: Typical immunofluorescence images. Right panel: Bar graphs summarizing the number of SIK2^+^ α-SMA^+^ cells in each field of view. Images were magnified by 200×. Data are presented as mean ± SD. * *p* < 0.05; ** *p* < 0.01; *** *p* < 0.001.

IPF serves as the most representative subtype of PF. To investigate the role of SIK2 in the pathogenesis of PF, we initially examined the protein levels of SIK2 in lung tissues obtained from IPF patients. Our findings indicated a significant upregulation of fibrotic markers, specifically fibronectin and α-SMA, in the lung tissues of IPF patients, accompanied by a marked 2.48-fold increase in SIK2 protein levels (*p* < 0.05) (Figure 1A). Subsequently, we assessed changes in SIK2 protein levels in the BLM-induced PF mouse model. Through the analysis of lung tissues from mice at various time points (0, 7, 14, and 21 days) post BLM administration, we observed a correlation between the progression of PF and the gradual increase in the expression levels of fibrotic markers alongside a concomitant 2.15-fold rise in SIK2 protein levels (*p* < 0.05) (Figure 1B). These results suggest that the protein level of SIK2 is elevated in both human and mouse models of PF, indicating a potential association between SIK2 and the occurrence of PF.

The lung tissue comprises a diverse array of cell types, including alveolar epithelial cells, endothelial cells, fibroblasts, smooth muscle cells, club cells, basal cells, and various immune cells such as macrophages. Among these, alveolar epithelial cells, fibroblasts, and macrophages play a central role in the pathogenesis of PF. Following the observation of elevated SIK2 protein expression in PF, it is imperative to ascertain the specific cell types in which this increase is predominantly localized. To elucidate the cellular localization of SIK2 within lung tissue, we conducted immunofluorescence co-staining of SIK2 with α-SMA (a myofibroblast marker) [26], SPC (a type II alveolar epithelial cell marker), and CD68 (a monocyte or macrophage marker) on lung tissue sections from patients with PF, including IPF and CTD-ILD. The findings demonstrated that SIK2 expression was elevated in the lung tissue of patients with IPF and CTD-ILD compared to healthy controls, with predominant co-localization observed with α-SMA but not with SPC or CD68 (Figure 1C and Figure S2). A similar pattern was observed in lung tissue sections from mice with BLM-induced PF, where SIK2 expression was elevated and co-localized with α-SMA (Figure 1D). These results suggest that in both PF mouse models and human patients, SIK2 is predominantly expressed in the increased population of myofibroblasts.

### 3.2. Fibroblast-Specific Sik2 Knockout Alleviates BLM-Induced Pulmonary Fibrosis in Mice

**Figure 2:**
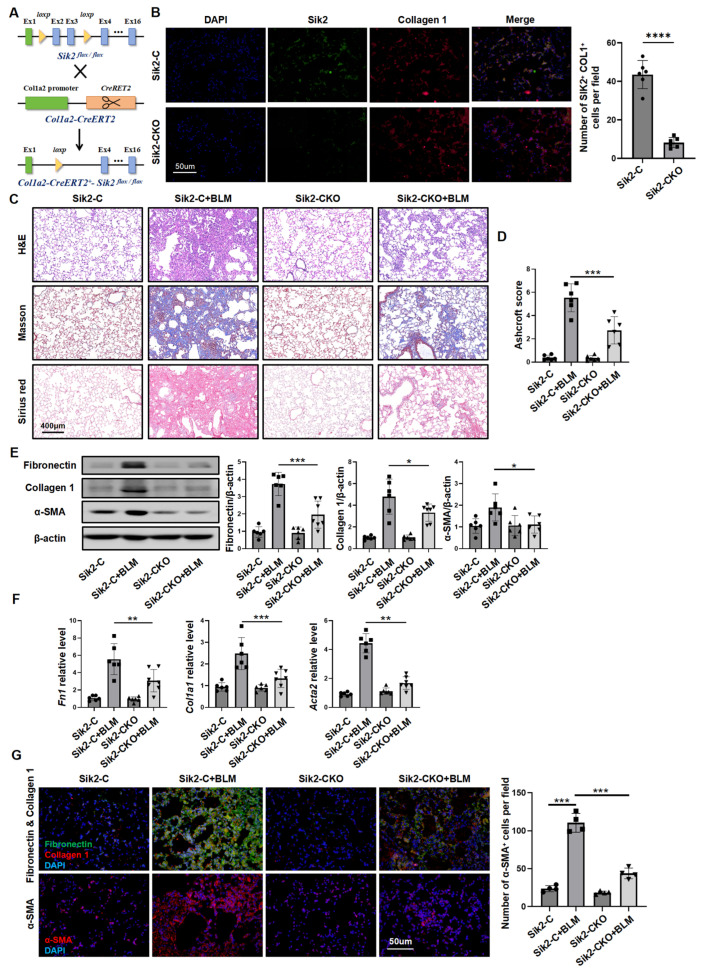
Fibroblast-specific *Sik2* knockout alleviates BLM-induced pulmonary fibrosis in mice. (**A**) A schematic diagram illustrating the construction strategy for generating transgenic mice utilized in this study. (**B**) Immunofluorescence co-staining of collagen1 and Sik2 was employed to evaluate the efficiency of *Sik2* knockout in fibroblasts derived from *Col1a2-Cre-Sik2^fl/fl^* mice, with images magnified at 200×. (**C**) Representative images of murine lung tissue sections stained with H&E, Masson’s Trichrome, and Sirius Red, captured at 50× magnification. (**D**) Ashcroft scoring for the assessment of fibrosis severity. (**E**) Analysis of collagen 1, α-SMA, and fibronectin protein levels in lung tissues from different groups of mice. Left panel: Representative Western blot images. Right panel: Bar graphs summarizing the expression levels of fibrotic markers across each group. Scale bar = 400 μm. (**F**) RT-qPCR results indicating the expression levels of *Col1a1*, *Acta2*, and *Fn1* in lung tissues from different groups of mice. (**G**) Immunofluorescence images depicting α-SMA, collagen 1, and fibronectin in lung tissue slices from mice, with images magnified at 200×. Left panel: Typical immunofluorescence images. Right panel: Bar graphs summarizing the number of α-SMA^+^ cells in each field of view. Sik2-CKO: *Col1a2-Cre-Sik2^fl/fl^* mice. Sik2-C: *Sik2^fl/fl^* mice. Data are presented as mean ± SD. * *p* < 0.05; ** *p* < 0.01; *** *p* < 0.001; **** *p* < 0.0001.

To clarify Sik2’s role in fibroblasts during PF, we generated fibroblast-specific *Sik2*-knockout mice (*Col1a2-CreERT2-Sik2^fl/fl^* mice) for our study (Figure 2A,B and Figure S3). Mice in the experimental groups were given BLM to induce PF, while the control group received normal saline. After 21 days, the mice were euthanized, and their lung tissues were collected to assess fibrosis severity. Histopathological analysis, including H&E staining and collagen-specific staining (Sirius Red and Masson’s trichrome staining), of lung tissue sections revealed that all mice subjected to BLM exhibited marked PF. However, *Sik2*-conditional-knockout (Sik2-CKO) mice had less fibrosis than *Sik2*-control (Sik2-C) mice, as confirmed by lower Ashcroft scores and hydroxyproline content (Figure 2C,D and Figure S4A). In Sik2-CKO mice, the expression of fibrosis markers (fibronectin, collagen 1, and α-SMA) and the number of α-SMA-positive cells in lung tissues were decreased by 60%, correlating with observed PF levels (Figure 2E–G). This indicates that knocking out *Sik2* in fibroblasts reduces myofibroblast numbers, potentially slowing PF progression by inhibiting fibroblast activation.

### 3.3. SIK2 Plays a Crucial Role in Fibroblasts Activation Process

**Figure 3:**
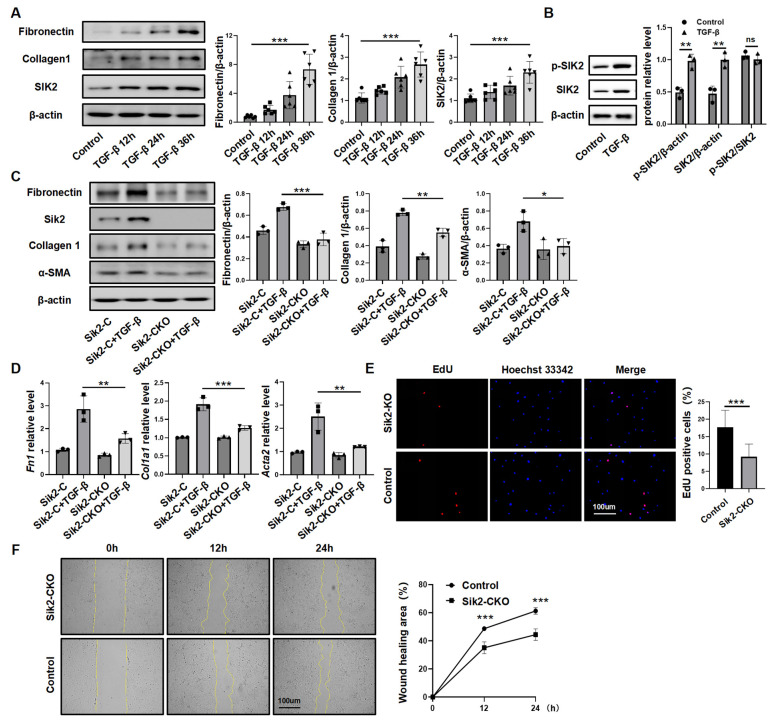
SIK2 plays a crucial role in mice lung fibroblasts activation process. (**A**) The protein levels of fibronectin, collagen 1, and SIK2 in the primary lung fibroblasts at different time points after TGF-β treatment. Left panel: Typical WB images. Right panel: Bar graphs summarizing the levels of each target across different groups. Each group includes six replicates. (**B**) The protein levels of phosphorylated SIK2 and total SIK2 in the primary lung fibroblasts after TGF-β treatment. Left panel: Typical WB images. Right panel: Bar graphs summarizing the levels of each target across different groups. Each group includes three replicates. The protein levels (**C**) and mRNA levels (**D**) of fibronectin, collagen 1, α-SMA, and SIK2 in the primary lung fibroblasts from Sik2-C mice or Sik2-CKO mice after TGF-β treatment. Each group includes three replicates. (**E**) EdU staining of the primary lung fibroblasts from Sik2-C mice or Sik2-CKO mice, shown in a representative image, with images magnified at 100×. Each group includes three replicates. Right panel: Bar graph displaying the results of the EdU assay. (**F**) Scratch assay to measure the migration capability of the primary lung fibroblasts from Sik2-C mice or Sik2-CKO mice, with images magnified at 100×. Left panel: Representative images showing wound healing progress at 0, 12, and 24 h. Right panel: Bar graph depicting the percentage of wound healing. Each group includes three replicates. Sik2-CKO: *Col1a2-Cre-Sik2^fl/fl^* mice. Sik2-C: *Sik2^fl/fl^* mice. Data are presented as mean ± SD. * *p* < 0.05; ** *p* < 0.01; *** *p* < 0.001; ns, no significance.

To examine the link between SIK2 and fibroblast activation, we studied SIK2 protein expression in vitro. Human lung fibroblasts (HPFs) were treated with TGF-β, and measurements were taken over time. Results showed that extended TGF-β exposure increased fibrotic markers and SIK2 levels, while the TGF-β receptor inhibitor SB431542 blocked these increases, suggesting that SIK2 expression rises during fibroblast activation (Figure 3A and Figure S5). Considering that SIK2 functions as a kinase and exists in a phosphorylated state that is intricately associated with its kinase activity, we investigated the alterations in phosphorylated SIK2 protein levels in TGF-β-stimulated HPFs. The results demonstrated a significant increase in the levels of phosphorylated SIK2 protein following TGF-β stimulation. However, the extent of this increase was not significantly different from that of the total SIK2 protein levels, indicating that while the total SIK2 protein levels rise during fibroblast activation, the degree of phosphorylation remains relatively stable (Figure 3B).

**Figure 4:**
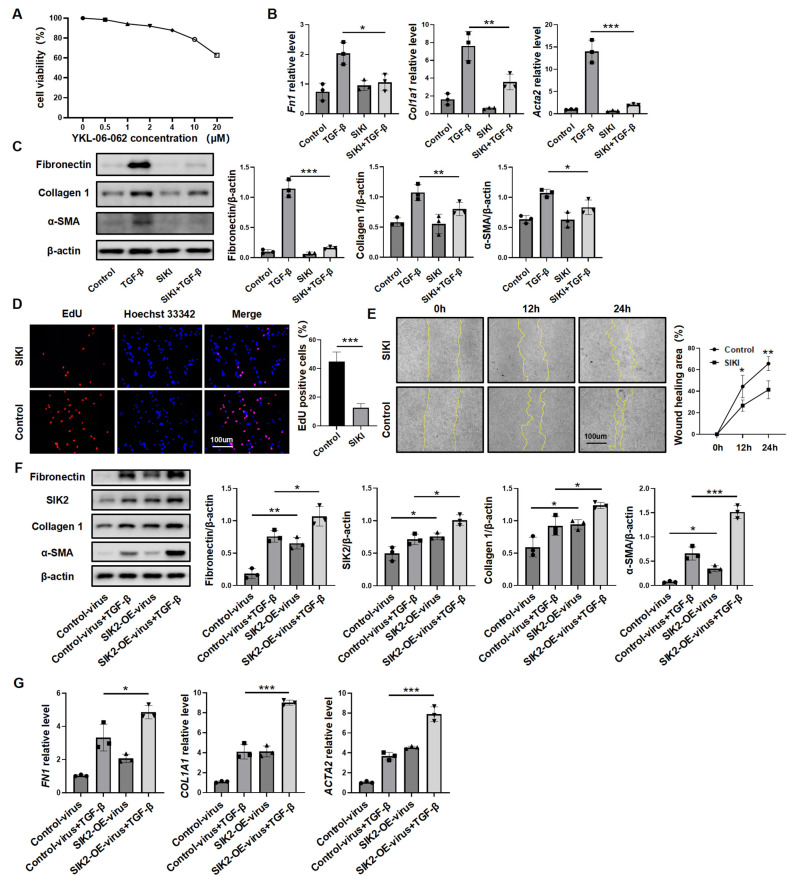
SIK2 also plays a crucial role in human lung fibroblasts activation process. (**A**) CCK8 assay to determine the impact of YKL-06-062 on human primary lung fibroblasts (HPFs) viability. Each concentration includes three replicates. The mRNA levels (**B**) and protein levels (**C**) of fibronectin, collagen 1, α-SMA, and SIK2 in HPFs treated with TGF-β (10 ng/mL) and YKL-06-062 (2 μM). SIKI: SIK2 inhibitor, YKL-06-062. Each group includes three replicates. (**D**) EdU staining of the HPFs treated with YKL-06-062 (2 μM), shown in a representative image, with images magnified at 100×. Each group includes three replicates. Right panel: Bar graph displaying the results of the EdU assay. (**E**) Scratch assay to measure the migration capability of the HPFs treated with YKL-06-062 (2 μM), with images magnified at 100×. Left panel: Representative images showing wound healing progress at 0, 12, and 24 h. Right panel: Bar graph depicting the percentage of wound healing. Each group includes three replicates. The protein levels (**F**) and mRNA levels (**G**) of fibronectin, collagen 1, α-SMA, and SIK2 in HPFs treated with TGF-β (10 ng/mL) and SIK2-overexpression virus. Each group includes three replicates. SIK2-OE: SIK2 overexpression. Data are presented as mean ± SD. * *p* < 0.05; ** *p* < 0.01; *** *p* < 0.001.

**Figure 5:**
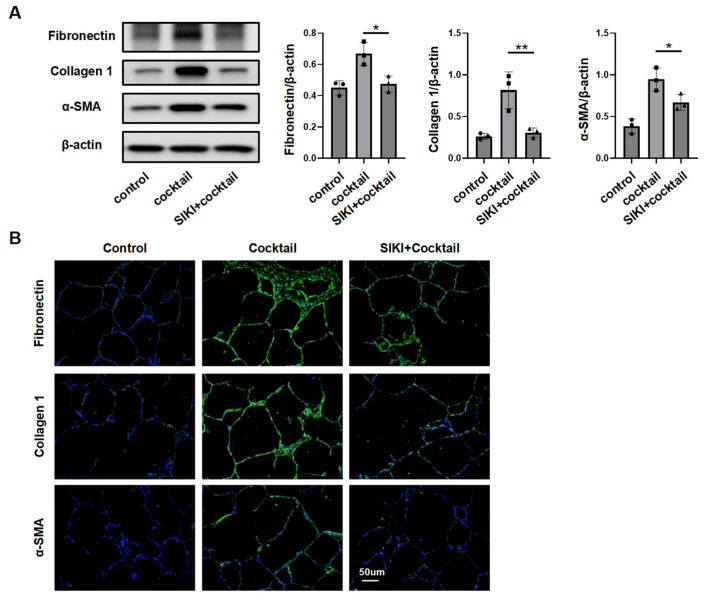
SIK2 inhibition alleviates fibrosis in PCLS. (**A**) Levels of fibrotic markers in hPCLS treated with a fibrosis-inducing cocktail and YKL-06-062 (2 μM) for 48 h, tested by Western blot. Each group includes three replicates. (**B**) Immunofluorescence staining of fibrotic markers in PCLS stimulated with the fibrosis-inducing cocktail and YKL-06-062 (2 μM) for 48 h, taken at a magnification of 200×. SIKI: SIK2 inhibitor, YKL-06-062. Data are presented as mean ± SD. * *p* < 0.05; ** *p* < 0.01.

To study Sik2 protein’s role, primary lung fibroblasts from *Sik2*-knockout mice were analyzed. Upon stimulation with TGF-β, fibroblasts derived from Sik2-C mice exhibited a significant upregulation in the expression levels of extracellular matrix components (fibronectin and collagen 1) as well as the molecular marker of fibroblast activation, α-SMA. Conversely, fibroblasts from Sik2-CKO mice did not demonstrate a statistically significant increase in the expression levels of these fibrosis markers following TGF-β stimulation (Figure 3C,D). During the activation of fibroblasts into myofibroblasts, there was not only an upregulation of fibrosis marker expression within the cells, but fibroblasts also exhibited enhanced proliferative and migratory capacities. Consequently, to provide a more comprehensive understanding of the impact of Sik2 on fibroblast functions, we also assessed these two functional attributes of fibroblasts. The scratch assay showed that TGF-β-stimulated *Sik2*-knockout mouse lung fibroblasts had significantly lower migration rates at 12 and 24 h compared to non-knockout cells (decreased by 28% both at 12 and 24 h) (Figure 3E). EdU staining and total number counts revealed that *Sik2* deletion significantly decreased the proportion of proliferating cells in primary mouse lung fibroblasts to 52% (Figure 3F and Figure S6). In addition, we treated HPFs with the SIK2 inhibitor YKL-06-062, finding it effectively prevents their activation into myofibroblasts and reduces their proliferation and migration, aligning with results from mouse cells (Figure 4A–E). Conversely, SIK2 overexpression increases fibrosis marker expression in HPFs and intensifies activation under TGF-β stimulation. Remarkably, even without TGF-β, SIK2 overexpression alone boosts fibrosis marker levels, showing its effect is not dependent on the presence of TGF-β (Figure 4F,G). In vitro cell experiments lack the tissue microenvironment and intercellular interactions seen in the lungs of patients. To better mimic human tissues, we used human precision-cut lung slices (HPCLS) to test if SIK2 inhibition remains effective against fibrosis. Our results showed that SIK2 inhibitors reduced fibrosis markers in these lung slices, indicating that SIK2 inhibition can effectively mitigate PF in a model that closely resembles the in vivo human condition (Figure 5A,B). In summary, SIK2 deficiency reduces fibrosis markers and inhibits proliferation and migration in mouse lung fibroblasts, highlighting SIK2’s essential role in fibroblast activation.

### 3.4. SIK2 Plays a Significant Role in the Regulation of Fibroblast Glucose Metabolism

**Figure 6:**
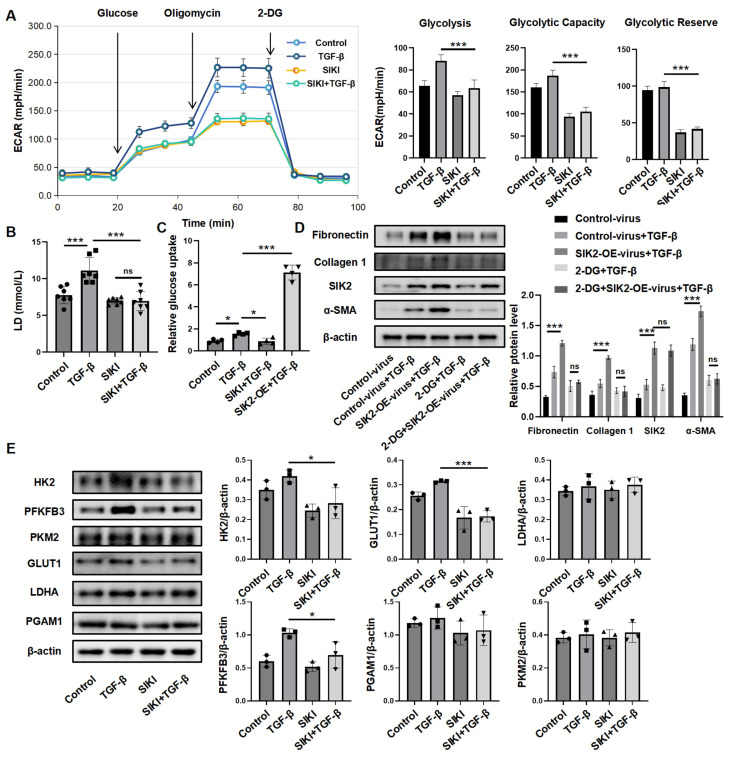
SIK2 plays a significant role in the regulation of fibroblast glucose metabolism. (**A**) The Seahorse glycolysis stress assay was used to examine the changes in the glycolytic levels of HPFs under the stimulation of TGF-β and YKL-06-062. Left panel: Graphs showing the ECAR values of each group of cells. Right panel: Statistical charts of glycolysis, glycolytic capacity, and glycolytic reserve for each group. Each group includes five replicates. (**B**) The detection results of lactic acid content in the cell culture supernatant of HPFs after stimulation with TGF-β and YKL-06-062 for 48 h. Each group includes seven replicates. (**C**) The results of the detection of the glucose uptake ability of HPFs 24 h after treatment with TGF-β, YKL-06-062 and SIK2 overexpression viruses. Each group includes four replicates. (**D**) The protein levels of fibronectin, collagen 1, α-SMA, and SIK2 in HPFs treated with TGF-β (10 ng/mL), 2-DG (2 mM), and YKL-06-062 (2 μM). Left panel: Typical WB images. Right panel: Bar graphs summarizing the levels of each target across different groups. Each group includes three replicates. (**E**) The protein levels of HK2, PFKFB3, PKM2, GLUT1, LDHA, and PGAM1 in HPFs treated with TGF-β (10 ng/mL) and YKL-06-062 (2 μM). Left panel: Typical WB images. Right panel: Bar graphs summarizing the levels of each target across different groups. Each group includes three replicates. SIKI: SIK2 inhibitor, YKL-06-062. SIK2-OE: SIK2 overexpression. Data are presented as mean ± SD. * *p* < 0.05; *** *p* < 0.001; ns, no significance.

Building on SIK2’s role in glucose metabolism in cancer, we hypothesized its similar influence in fibroblasts. To examined how SIK2 affects glucose metabolism, we employed the seahorse glycolysis stress test to measure the glucose metabolism level of HPFs. Results showed that TGF-β increased glycolysis and glycolytic capacity, while SIK2 inhibitor significantly reduced both, along with glycolytic reserve (Figure 6A). This indicates that SIK2 inhibition greatly diminishes fibroblasts’ glucose metabolic capacity. Lactic acid production during anaerobic glycolysis serves as an indicator of the intensity of glucose metabolism within cells. We measured lactate in the culture medium of human fibroblasts treated with TGF-β and the SIK2 inhibitor for 48 h. TGF-β treatment significantly increased lactic acid production, while the SIK2 inhibitor notably decreased it to 63%, suggesting that SIK2 inhibition reduces anaerobic glycolysis in fibroblasts (Figure 6B). In addition, we studied SIK2’s impact on the glucose uptake in HPFs and found that TGF-β treatment significantly increased uptake, while SIK2 inhibitors reduced it to 61%. Fibroblasts with SIK2 overexpression maintained 3.2-fold-higher glucose uptake than those treated with TGF-β alone, indicating SIK2 regulates this process (Figure 6C). To determine if SIK2’s enhancement of fibroblast activation relies on its influence on glucose metabolism, we used the glycolysis inhibitor 2-deoxy-D-glucose (2-DG) on SIK2-overexpressing fibroblasts treated with TGF-β. Results showed that 2-DG significantly reduced fibroblast activation levels regardless of SIK2 overexpression. No significant differences in fibrosis markers were observed between the 2-DG + SIK2-OE-virus + TGF-β group and the 2-DG + TGF-β group, indicating that 2-DG negated SIK2’s activation effect (Figure 6D). Thus, SIK2’s role in fibroblast activation depends on its regulation of glucose metabolism.

The regulation of glucose metabolism in fibroblasts is influenced by multiple factors. To elucidate the mechanism by which SIK2 modulates glucose metabolism in fibroblasts, we investigated the impact of SIK2 on the protein expression levels of enzymes associated with glucose metabolism. Our findings demonstrated that the inhibition of SIK2 led to a reduction in the expression levels of HK2, PFKFB3, and GLUT1 in fibroblasts, whereas the expression levels of PKM2, LDHA, and PGAM1 were unaffected (Figure 6E). Both HK2 and PFKFB3 are pivotal enzymes in the glycolytic pathway, and their expression levels are crucial determinants of the cellular glycolytic rate. GLUT1, responsible for glucose transport, significantly influences the cellular glucose uptake capacity. These results suggest that SIK2 enhances glucose metabolism in fibroblasts by upregulating the expression of glycolysis-related enzymes HK2, PFKFB3, and GLUT1.

### 3.5. Inhibiting SIK2 Is a Promising Approach for Anti-Fibrosis

**Figure 7:**
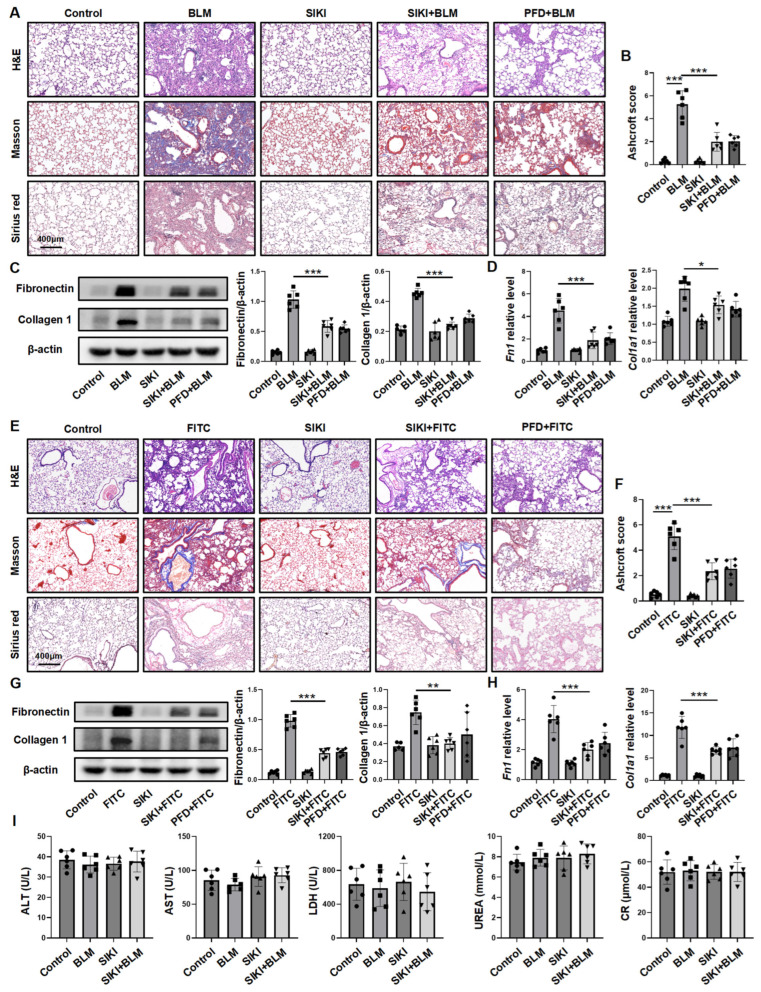
The SIK2 inhibitor YKL-06-062 can inhibit pulmonary fibrosis in mouse models of pulmonary fibrosis induced by BLM or FITC. (**A**) Intervention in the mouse pulmonary fibrosis induced by BLM (2 mg/kg) using YKL-06-062 (2 mg/kg) or pirfenidone (PFD, 200 mg/kg, as a positive control). Representative images of murine lung tissue sections stained with H&E, Masson’s Trichrome, and Sirius Red, captured at 50× magnification. (**B**) Ashcroft scoring for the assessment of fibrosis severity; The protein levels (**C**) and mRNA levels (**D**) of fibronectin and collagen 1 in the lung tissues of each group of mice. (**E**) Intervention in the mouse pulmonary fibrosis induced by FITC (20 mg/kg) using YKL-06-062 (2 mg/kg) or pirfenidone (PFD, 200 mg/kg, as a positive control). Representative images of murine lung tissue sections stained with H&E, Masson’s Trichrome, and Sirius Red, captured at 50× magnification. (**F**) Ashcroft scoring for the assessment of fibrosis severity. The protein levels (**G**) and mRNA levels (**H**) of fibronectin and collagen 1 in the lung tissues of each group of mice; (**I**) alanine aminotransferase (ALT), aspartate aminotransferase (AST), lactate dehydrogenase (LDH), urea (UREA), and creatinine (Cr) levels in mouse serum across each group. The statistical data reflect findings from six mice in each group, presented as mean ± SD. SIKI: SIK2 inhibitor, YKL-06-062. ALT: alanine aminotransferase. AST: aspartate aminotransferase. Cr: creatinine. LDH: lactate dehydrogenase. * *p* < 0.05; ** *p* < 0.01; *** *p* < 0.001.

To assess the effectiveness of the SIK2 inhibitor YKL-06-062 in reducing PF, we treated the BLM-induced PF mouse model with YKL-06-062. Histopathological and collagen staining as well as hydroxyproline content showed severe fibrosis in BLM-induced mice, while those treated with YKL-06-062 had significantly less fibrosis (Figure 7A and Figure S4B). The Ashcroft score confirmed these results, showing less fibrosis in the YKL-06-062+BLM group compared to the BLM group (Figure 7B). Western blot and RT-qPCR analyses revealed reduced expression of fibrosis markers in the YKL-06-062-treated mice (Figure 7C,D). To further assess YKL-06-062’s effectiveness against PF, we used an FITC-induced PF mouse model. Similar to results from the BLM model, lung tissue analysis showed significantly reduced fibrosis in YKL-06-062-treated mice compared to untreated ones (Figure 7E–H and Figure S4C), confirming YKL-06-062’s strong anti-fibrotic effects in both mouse models. A comparative analysis demonstrated that the anti-fibrotic efficacy of intraperitoneally administered YKL-06-062 at a dosage of 2 mg/kg on alternate days is comparable to that of daily oral administration of pirfenidone at a dosage of 200 mg/kg. Blood tests in mice indicated no significant liver or kidney function changes, thereby providing preliminary evidence of YKL-06-062’s safety (Figure 7I). Overall, YKL-06-062 effectively reduces PF in mouse models, indicating its potential as a treatment.

**Figure 8:**
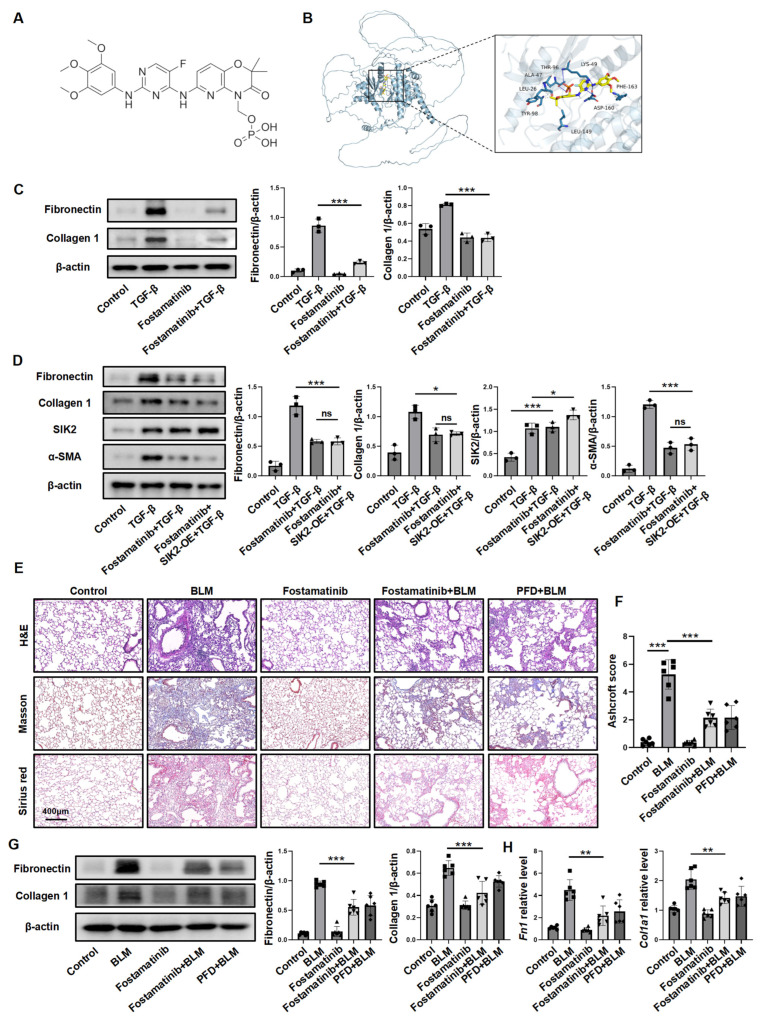
Fostamatinib exerts anti-fibrotic effects by inhibiting SIK2, representing a potential option for clinical anti-fibrotic treatment. (**A**) Structural representation of fostamatinib. (**B**) Molecular docking diagram of fostamatinib with SIK2 protein. (**C**) The protein levels of fibronectin and collagen 1 in the HPFs treated with TGF-β (10 ng/mL) and fostamatinib (20 μM). Left panel: Typical WB images. Right panel: Bar graphs summarizing the levels of each target across different groups. Each group includes three replicates. (**D**) The protein levels of fibronectin, collagen 1, SIK2, and α-SMA in the HPFs treated with SIK2 overexpression virus, TGF-β (10 ng/mL), and fostamatinib (20 μM). Left panel: Typical WB images. Right panel: Bar graphs summarizing the levels of each target across different groups. Each group includes three replicates. (**E**) Intervention in the mouse pulmonary fibrosis induced by BLM (2 mg/kg) using fostamatinib (20 mg/kg) or pirfenidone (PFD, 200 mg/kg). Representative images of murine lung tissue sections stained with H&E, Masson’s Trichrome, and Sirius Red, captured at 50× magnification. (**F**) Ashcroft scoring for the assessment of fibrosis severity. The protein levels (**G**) and mRNA levels (**H**) of fibronectin and collagen 1 in the lung tissues of each group of mice. The statistical data reflect findings from six mice in each group, presented as mean ± SD. * *p* < 0.05; ** *p* < 0.01; *** *p* < 0.001; ns, no significance.

Small-molecule kinase inhibitors are extensively utilized in clinical practice, with numerous such inhibitors having received FDA approval for the treatment of various diseases. We hypothesize that among these clinically employed small-molecule kinase inhibitors, there may exist compounds capable of inhibiting SIK2. To investigate this possibility, we employed computational molecular docking simulations to predict the binding affinity of these small molecules to the SIK2 protein. Our findings indicated that fostamatinib, a drug initially approved for the treatment of chronic thrombocytopenia, was predicted to bind to the SIK2 protein (Figure 8A,B). Next, we tested fostamatinib’s ability to inhibit fibroblast activation by examining its impact on TGF-β-induced fibroblasts, and the results showed that fostamatinib significantly reduced extracellular matrix production (Figure 8C). Furthermore, fostamatinib completely blocked the activation-enhancing effect of SIK2 overexpression in fibroblasts, indicating that it inhibited the function of SIK2 (Figure 8D). Following that, we proceeded to assess its potential anti-fibrotic effects in vivo using a BLM-induced PF mouse model. Histopathological analysis and hydroxyproline content demonstrated a significant less fibrosis in the fostamatinib + BLM group compared to the BLM group (Figure 8E,F and Figure S4D). Furthermore, evaluations employing Western blotting, RT-qPCR, and immunofluorescence staining for fibrotic markers consistently indicated decreased expression levels of fibrotic markers in the fostamatinib-treated group, correlating with a reduction in the severity of PF (Figure 8G,H and Figure S7). In summary, fostamatinib inhibits SIK2, preventing fibroblast activation and reducing PF in mice, suggesting its potential for clinical application in the treatment of PF.

## 4. Discussion

PF constitutes a range of disorders characterized by the development of fibrotic foci within the lung parenchyma, which can obstruct normal gas exchange, leading to symptoms such as dyspnea and coughing and, in severe instances, progressing to respiratory failure and mortality [27]. This study elucidates the mechanistic role of salt-inducible kinase 2 (SIK2) as a pivotal contributor to the progression of pulmonary fibrosis, primarily through the activation of glycolysis. Additionally, it demonstrates the efficacy and preliminary safety of selectively targeting SIK2 using the inhibitor YKL-06-062 and the repurposed agent fostamatinib in mitigating pulmonary fibrosis in vivo. These findings offer a crucial mechanistic foundation and proof-of-concept for the development of SIK2-targeted antifibrotic strategies.

Nintedanib, one of the two FDA-approved therapeutic agents for pulmonary fibrosis (PF), is well-characterized for its broad-spectrum inhibition of VEGFR, PDGFR, and FGFR. Notably, existing studies suggest that nintedanib may also exert off-target inhibitory effects against SIK2. To date, several small-molecule inhibitors targeting SIK2 have been reported, including ARN-3236, YKL-05-099, and GLPG3970 [28]. Previous work by Jiang et al. utilized ARN-3236 to demonstrate the anti-fibrotic activity of SIK2 inhibition in both in vitro and in vivo models [23]. Similarly, Arthur and colleagues employed a whole-body SIK2-inactive-knockin mouse model to reveal that SIK2 loss-of-function ameliorates the pathological progression of PF [29]. However, these prior studies possess significant limitations: First, pharmacological approaches inherently struggle to fully exclude potential off-target effects of the inhibitors used. Second, whole-body genetic intervention mouse models lack cell type-specificity in target perturbation, resulting in imprecise mechanistic interpretation. In this study, we employed fibroblast-specific *Sik2*-knockout mice to investigate the role of SIK2 in fibroblasts concerning the progression of PF. By utilizing highly specific gene-editing techniques, we obtained definitive evidence regarding cell type specificity and gene target specificity.

HPCLS is a novel organoid model prepared from fresh human or experimental animal lung tissues with biological activity [30,31]. These sections mimic the in vivo cellular interactions and microenvironment, preserving the 3D structure, cell diversity, and extracellular matrix of lung tissue. This organoid model offers a more accurate representation of the in vivo environment compared to isolated cell cultures. In this study, primary lung fibroblasts helped reveal SIK2’s role in fibroblasts, but they did not capture the complex environment of PF. To more precisely replicate the clinical conditions of patients, we investigated the effects of SIK2 inhibition using HPCLS. The results demonstrated that the application of SIK2 inhibitors in these sections effectively inhibited fibroblast activation and reduced extracellular matrix production, thereby underscoring the potential of anti-SIK2 therapy for clinical application.

The Warburg effect, initially identified in cancer cells, describes the preferential utilization of anaerobic glycolysis for energy production despite the presence of abundant oxygen. This metabolic strategy not only facilitates rapid energy generation but also supplies substrates necessary for the biosynthesis of ribose, amino acids, and other macromolecules while concurrently producing lactic acid, thereby contributing to the formation of an acidic microenvironment [32]. These factors collectively support the vigorous proliferation and metabolic activity characteristic of cancer cells. A similar metabolic phenomenon is observed in other cell types; for instance, during fibroblast activation, the upregulation of glycolytic pathways underpins their enhanced capacity for extracellular matrix secretion, proliferation, and migration. In our study, we revealed that SIK2 significantly influenced the expression levels of three critical enzymes: HK2, GLUT1, and PFKFB3. GLUT1 is an enzyme responsible for transporting glucose into cells, and its expression level is related to the ability of cells to take up glucose [33]. This connection between SIK2 and GLUT1 also explains the promoting effect of SIK2 overexpression on the glucose uptake ability of fibroblasts. HK2 is an enzyme that catalyzes the first step of glycolysis and is one of the key enzymes of glycolysis, responsible for fixing glucose in cells through phosphorylation and initiating the glycolysis reaction [34]. PFKFB3 is also one of the key enzymes of glycolysis and can catalyze fructose-6-phosphate into fructose-2,6-diphosphate to activate phosphofructokinase 1 and enhance the glycolysis level of cells [15]. These three enzymes play a crucial role in cellular glycolysis, and the influence of SIK2 on their expression levels underscores its significant regulatory impact on this metabolic pathway.

One of the primary objectives of this study is to investigate the feasibility of SIK2-targeted anti-fibrotic therapeutic strategies. Therapeutic strategies targeting specific genetic elements include RNA interference (RNAi therapy), neutralizing antibody therapy, and small-molecule inhibitor therapy [35,36]. Among these, RNAi therapy has garnered significant attention and is the subject of numerous clinical trials; however, its clinical application remains limited. Neutralizing antibody therapy primarily targets secreted proteins or is employed in antiviral treatments. Small-molecule inhibitors, on the other hand, are widely utilized due to their advantages, such as convenient oral administration, strong permeability, high efficacy, and relatively low cost [37]. Notably, the two most prominent anti-fibrotic drugs in clinical use, pirfenidone and nintedanib, are small molecules [38]. Consequently, this study focuses on employing small-molecule inhibitors targeting SIK2 as a potential intervention for PF. Several small-molecule inhibitors for SIK2 have been developed, with YKL-06-062 showing notable selectivity. We tested YKL-06-062 in mouse models of BLM or FITC-induced PF, and histopathology, collagen staining, as well as fibrotic marker levels confirmed its anti-fibrotic effects. In addition to using the SIK2 inhibitor YKL-06-062, we also hope to explore more intervention schemes targeting SIK2 to treat PF. Currently, dozens of small-molecule kinase inhibitors have been approved for use in clinical practice by the FDA [39]. These small-molecule kinase inhibitors exert their inhibitory effects by combining with their target kinases in various ways. SIK2 is also a kinase, and we speculate that there may be inhibitors among the kinase inhibitors already used in clinical practice that also have inhibitory effects on SIK2. Molecular docking is a computer-based method that predicts interactions between ligands and targets, useful for modeling how small molecules, proteins, or peptides bind [40]. We used it to screen small-molecule kinase inhibitors against SIK2 proteins. The simulations indicated that fostamatinib, a drug for chronic thrombocytopenia, could potentially bind to SIK2. We conducted cell experiments to test if fostamatinib inhibits fibroblasts by targeting SIK2 protein. Results showed that fostamatinib effectively blocked SIK2’s role in fibroblast activation, indicating its inhibitory effects depend on SIK2 suppression. We further confirmed fostamatinib’s anti-fibrotic effects in a BLM-induced PF mouse model. In clinical practice, the standard dosage of fostamatinib for the treatment of chronic thrombocytopenia is 100 mg administered twice daily [41]. When translating this dosage to a murine model, it equates to 35.2 mg/kg per day. However, in this study, a daily dose of 10 mg/kg was sufficient to alleviate PF in the mice, suggesting that administering only a low dose of fostamatinib is sufficient to elicit an anti-pulmonary fibrosis effect. Furthermore, a reduced dosage may minimize the risk of side effects and adverse reactions, thereby underscoring the potential of fostamatinib as a therapeutic agent for PF.

This study is subject to certain limitations. Firstly, while it identified that the SIK2 protein can regulate the expression levels of glycolysis-related enzymes such as HK2, GLUT1, and PFKFB3, the specific regulatory mechanisms remain unelucidated. Secondly, although the study suggests that fostamatinib holds promise as a novel option for clinical anti-fibrotic therapy, it does not comprehensively assess its safety and efficacy in clinical settings. These issues warrant further investigation.

In conclusion, this study highlights the role of SIK2 in promoting PF by enhancing fibroblast glycolysis through up-regulating glycolysis-related enzymes. The SIK2 inhibitor YKL-06-062 and fostamatinib were tested for effectiveness in mouse models, showing potential as a treatment for PF. These findings suggest that targeting SIK2 could be a promising clinical approach for treating PF.

## Data Availability

The original contributions presented in this study are included in the article/Supplementary Materials. Further inquiries can be directed to the corresponding author.

## Supplementary Materials

The following supporting information can be downloaded at: https://www.mdpi.com/article/10.3390/biomedicines13081919/s1, Figure S1: Schematic diagram of animal experiments; Figure S2: SIK2 is not expressed in macrophages or alveolar epithelial cells of patients with IPF; Figure S3: Genotyping results diagram of Col1a2-Cre mice and Sik2-loxp mice; Figure S4: The results of hydroxyproline content detection in animal experiments; Figure S5: The increase in SIK2 expression is dependent on the TGF-β pathway; Figure S6: Impact of YKL-06-062 on total cell counts; Figure S7: The immunofluorescence results of fostamatinib in alleviating pulmonary fibrosis in mice. Table S1: the baseline characteristics of participants who contributed lung tissue samples; Table S2: Primers for RT-qPCR.

## Abbreviations

BLM	bleomycin
DMEM	Dulbecco’s modified eagle medium
DMSO	dimethyl sulfoxide
FITC	fluorescein Isothiocyanate
HPCLS	human precision-cut lung slices
HPFs	human primary pulmonary fibroblasts
PDGF	platelet-derived growth factor
PF	pulmonary fibrosis
PFD	pirfenidone
TGF-β	transforming growth factor-beta
TNF-α	tumor necrosis factor-alpha
2-DG	2-deoxy-D-glucose
