# Supplementary material for: SIK2 Drives Pulmonary Fibrosis by Enhancing Fibroblast Glycolysis and Activation
Source: Biomedicines. 2025 Aug 6;13(8):1919. doi: 10.3390/biomedicines13081919 (PMC12383515; doi:10.3390/biomedicines13081919)
Supplement: Supplementary file 1 [file biomedicines-13-01919-s001.zip › biomedicines-3746747-supplementary.pdf]

**Supplementary Table 1. Baseline characteristics of the study participants who provided lung tissue samples**

| Basic characteristics | Control Subjects (n=5) | IPF Subjects (n=5) | CTD-ILD Subjects (n=1) |
|---|---|---|---|
| **Age, years** | | | |
| mean (SD) | 44.7 (9.7) | 47.1 (9.6) | 51.5 (3.5) |
| **Gender** | | | |
| Female | 2 (40%) | 1 (20%) | 0 |
| Male | 3 (60%) | 4 (80%) | 2 (100%) |

**Supplementary Table 2: Primers for RT-qPCR**

| | | |
|---|---|---|
| human *FN1* | forward | 5′-AGGAAGCCGAGGTTTTAACTG-3′ |
| | reverse | 5′-AGGACGCTCATAAGTGTCACC-3′ |
| human *COL1A1* | forward | 5′-GAGGGCCAAGACGAAGACATC-3′ |
| | reverse | 5′-CAGATCACGTCATCGCACAAC-3′ |
| human *ACTA2* | forward | 5′-AAAAGACAGCTACGTGGGTGA-3′ |
| | reverse | 5′-GCCATGTTCTATCGGGTACTTC-3′ |
| human *ACTB* | forward | 5′-CATGTACGTTGCTATCCAGGC-3′ |
| | reverse | 5′-CTCCTTAATGTCACGCACGAT-3′ |
| human *SIK2* | forward | 5′-AGACCACCCTCACATAATCAAAC-3′ |
| | reverse | 5′-ATTTTCGCCTGGCTTCAGACT-3′ |
| mouse *Fn1* | forward | 5′-GATGTCCGAACAGCTATTTACCA-3′ |
| | reverse | 5′-CCTTGCGACTTCAGCCACT-3′ |
| mouse *Col1a1* | forward | 5′-TAAGGGTCCCCAATGGTGAGA-3′ |
| | reverse | 5′-GGGTCCCTCGACTCCTACAT-3′ |
| mouse *Acta2* | forward | 5′-GTCCCAGACATCAGGGAGTAA-3′ |
| | reverse | 5′-TCGGATACTTCAGCGTCAGGA-3′ |
| mouse *Actb* | forward | 5′-GGCTGTATTCCCCTCCATCG-3′ |
| | reverse | 5′-CCAGTTGGTAACAATGCCATGT-3′ |
| mouse *Sik2* | forward | 5′-CTGCTGGCAACATGGTGTG-3′ |
| | reverse | 5′-GGGAGAGTTGGTCCATCAAAAG-3′ |

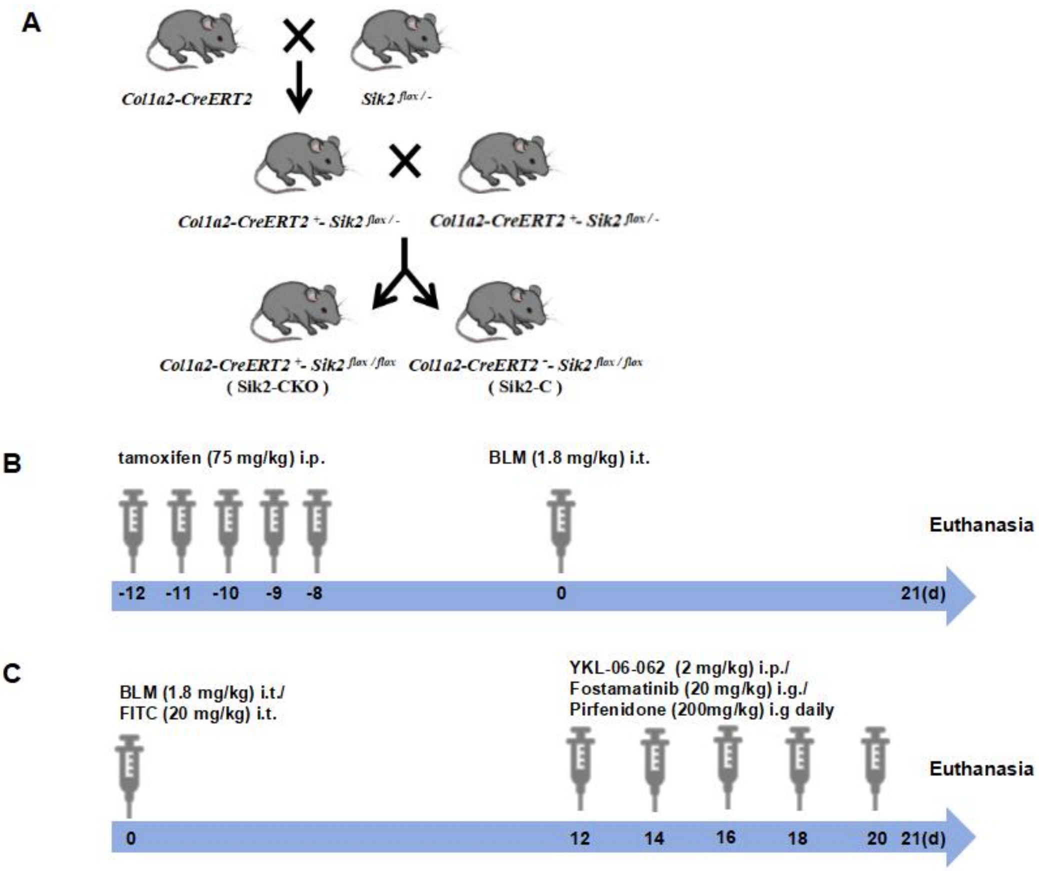


**Supplementary figure 1.**

A. Breeding strategy for Col1a2-Cre-Sik2fl/fl mice

B. Experimental timeline for conditional Sik2 knockout and pulmonary fibrosis induction

C. Drug administration protocol for small-molecule inhibitors

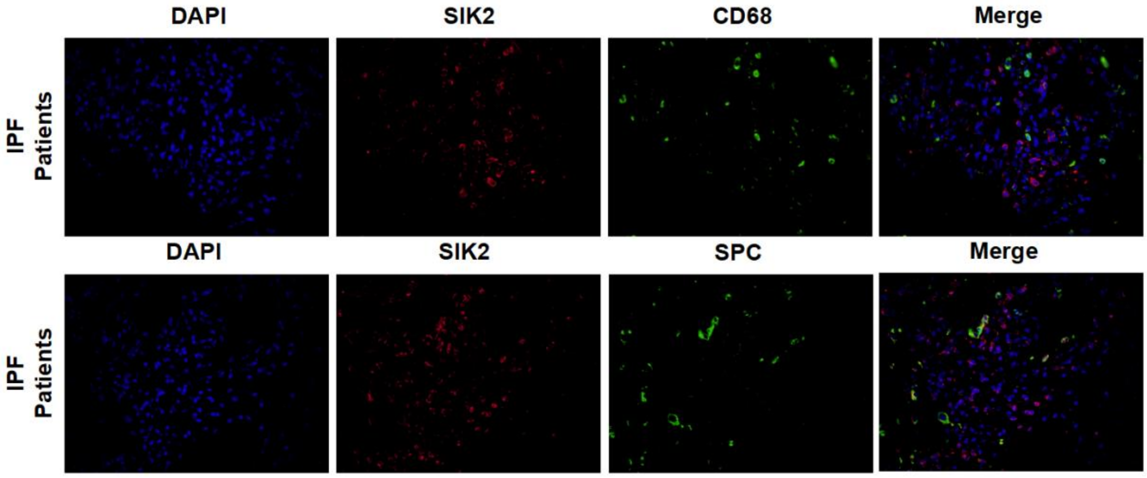


**Supplementary Figure 2.**

Immunofluorescence images of SIK2, CD68, and SPC in lung tissue slices of IPF patients.

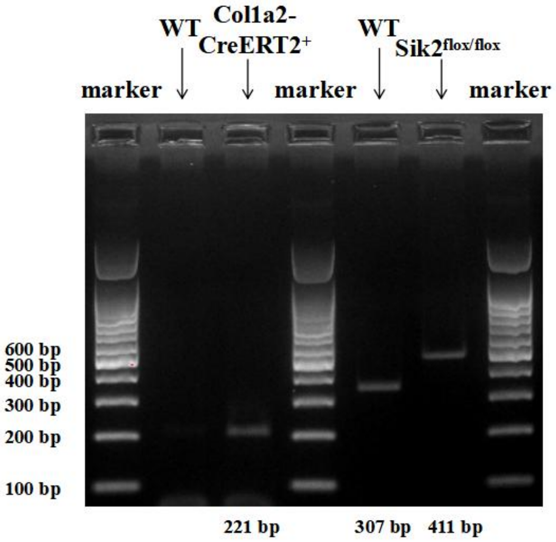


**Supplementary Figure 3.**

Genotyping results diagram of *Col1a2-Cre* mice and *Sik2-loxp* mice.

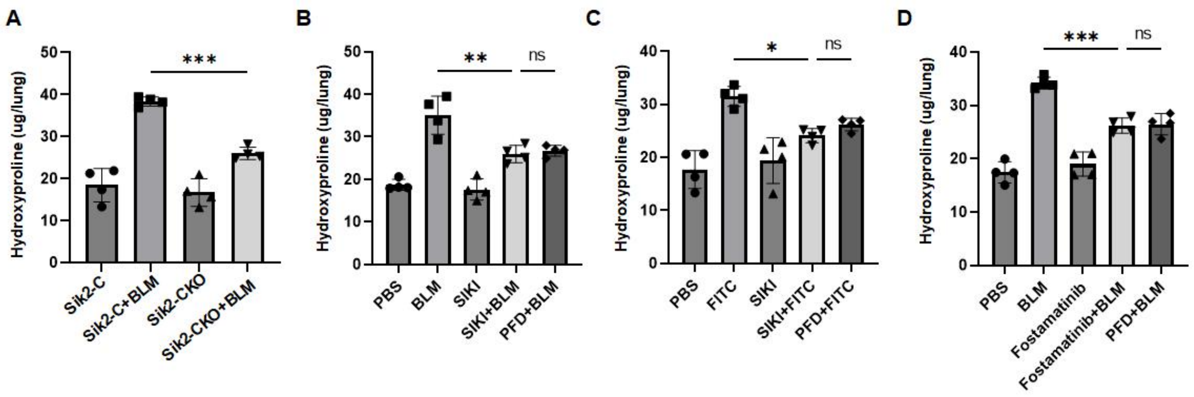


**Supplementary figure 4.**

A. Lung hydroxyproline content in Col1a2-Cre-Sik2fl/fl mice following bleomycin challenge

B. Effect of YKL-06-062 (2 mg/kg) or pirfenidone (PFD, 200 mg/kg) treatment on hydroxyproline levels in bleomycin-induced pulmonary fibrosis (2 mg/kg)

C. Effect of YKL-06-062 (2 mg/kg) or pirfenidone (PFD, 200 mg/kg) treatment on hydroxyproline levels in FITC-induced pulmonary fibrosis (20 mg/kg)

D. Effect of fostamatinib (20 mg/kg) or pirfenidone (PFD, 200 mg/kg) treatment on hydroxyproline levels in bleomycin-induced pulmonary fibrosis (2 mg/kg)

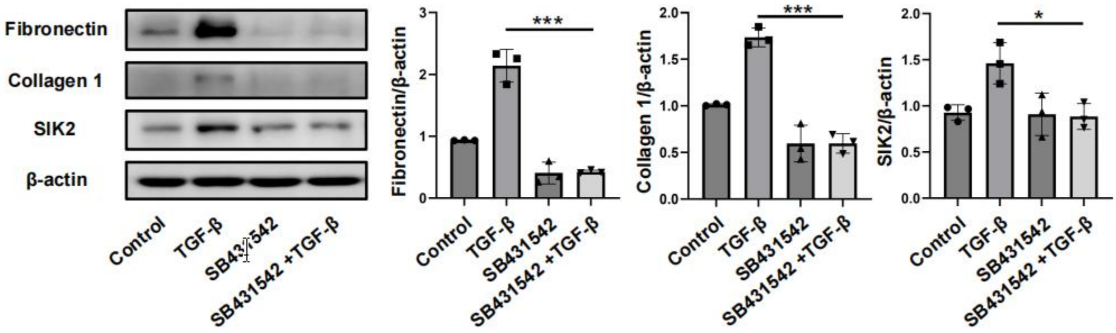


**Supplementary Figure 5.**

The protein levels of Fibronection, Collagen 1, and SIK2 in the HPFs treated with TGF-β (10 ng/mL) and SB431542 (2 μM). Left panel: Typical WB images. Right panel: Bar graphs summarizing the levels of each target across different groups. Data are presented as mean ± SD. *$p$<0.05; *$p$<0.05; **$p$<0.01.

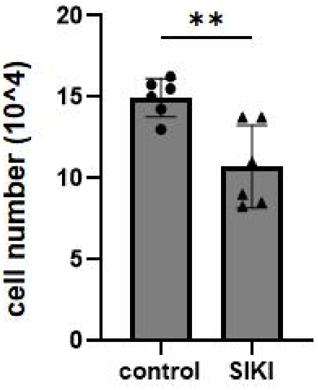


**Supplementary figure 6**

Impact of YKL-06-062 on total cell counts

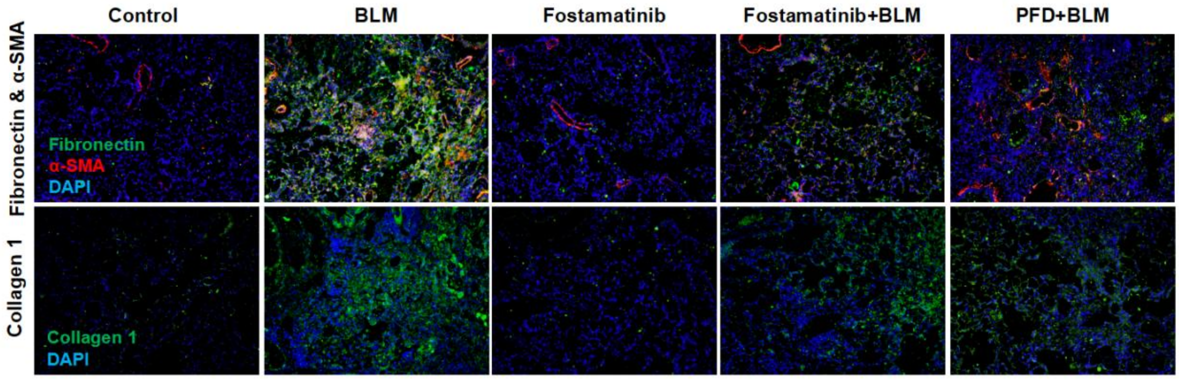


**Supplementary Figure 7.**

Intervene in the mouse pulmonary fibrosis induced by BLM (2 mg/kg) using fostamatinib (20 mg/kg) or pirfenidone (PFD, 200 mg/kg). Immunofluorescence images of α-SMA, Collagen 1, and Fibronectin in mice lung tissue slices. Images were magnified by 200×.
